# Suppression of Lung Tumorigenesis by Leucine Zipper/EF Hand–Containing Transmembrane-1

**DOI:** 10.1371/journal.pone.0012535

**Published:** 2010-09-02

**Authors:** Soon-Kyung Hwang, Longzhen Piao, Hwang-Tae Lim, Arash Minai-Tehrani, Kyeong-Nam Yu, Youn-Cheol Ha, Chan-Hee Chae, Kee-Ho Lee, George R. Beck, Jongsun Park, Myung-Haing Cho

**Affiliations:** 1 Laboratory of Toxicology, College of Veterinary Medicine, Seoul National University, Seoul, Korea; 2 Nano Systems Institute-National Core Research Center, Seoul National University, Seoul, Korea; 3 Graduate Group of Tumor Biology, Seoul National University, Seoul, Korea; 4 Department of Oncology, Affiliated Hospital of Yanbian University, Jilin, China; 5 Department of Veterinary Pathology, College of Veterinary Medicine, Seoul National University, Seoul, Korea; 6 Laboratory of Radiation Molecular Oncology, Korea Institute of Radiological & Medical Sciences, Seoul, Korea; 7 Division of Endocrinology, Metabolism and Lipids, Emory University School of Medicine, Atlanta, Georgia, United States of America; 8 Department of Pharmacology, College of Medicine, Daejeon Regional Cancer Center, Cancer Research Institute, Research Institute for Medical Sciences, Chungnam National University, Daejeon, Korea; University of Barcelona, Spain

## Abstract

**Background:**

Leucine zipper/EF hand-containing transmembrane-1 (LETM1) encodes for the human homologue of yeast Mdm38p, which is a mitochondria-shaping protein of unclear function. However, a previous study demonstrated that LETM1 served as an anchor protein for complex formation between mitochondria and ribosome, and regulated mitochondrial biogenesis.

**Methodology/Principal Findings:**

Therefore, we examine the possibility that LETM1 may function to regulate mitochondria and lung tumor growth. In this study, we addressed this question by studying in the effect of adenovirus-mediated LETM1 in the lung cancer cell and lung cancer model mice. To investigate the effects of adenovirus-LETM1 *in vitro*, we infected with adenovirus-LETM1 in A549 cells. Additionally, *in vivo* effects of LETM1 were evaluated on K-*ras* ^LA1^ mice, human non-small cell lung cancer model mice, by delivering the LETM1 via aerosol through nose-only inhalation system. The effects of LETM1 on lung cancer growth and AMPK related signals were evaluated. Adenovirus-mediated overexpression of LETM1 could induce destruction of mitochondria of lung cancer cells through depleting ATP and AMPK activation. Furthermore, adenoviral-LETM1 also altered Akt signaling and inhibited the cell cycle while facilitating apoptosis. Theses results demonstrated that adenovirus-LETM1 suppressed lung cancer cell growth *in vitro* and *in vivo*.

**Conclusions/Significance:**

Adenovirus-mediated LETM1 may provide a useful target for designing lung tumor prevention and treatment.

## Introduction

Lung cancer is the leading cause of cancer deaths in the world with over one million cases diagnosed every year. Multiple options for the treatment of lung cancer have been described, including surgery, chemotherapy, and radiation, however, therapeutic effect is typically transient and mostly absent with advanced disease [1], [2]. Therefore, the need of more rational approach to lung cancer therapy is essential.

Recently, mitochondria have emerged as effective target for anti-cancer therapy [3]. Mitochondria are multifunctional organelles of changing morphology whose activities are intimately tied to cell physiology [4]. Fragmented mitochondrial morphology, for example, is correlated with apoptotic cytochrome *c* release, whereas tubular morphology promotes resistance to apoptotic stimuli. Also, mitochondria function as central components of cell survival through ATP production and govern cell fate by mitochondrial membrane-dependent cell death signal [5].

Cellular energy is largely derived from the process of oxidative phosphorylation in the mitochondria. The status of cellular energy stores is monitored by AMP-activated protein kinase (AMPK). AMPK is activated to reserve cellular energy content, and serves as a key regulator of cell survival or death in response to pathological stresses [6], [7]. Under conditions of ATP depletion, AMPK is allosterically activated by the binding of AMP to AMPK, which facilitates phosphorylation of AMPK on Threonine 172 [8], [9]. Since AMPK is associated with many critical cellular events, considerable amount of efforts has devoted to elucidate whether AMPK is implicated in cancer cell growth and metabolism. In fact, recent line of evidence suggest that the inactivation of AMPK augments malignant behaviors of prostate cancer cells and its activation suppresses their growth [10].

Cell cycle progression is intricately controlled under many checkpoints which respond to intrinsic and extrinsic signals. During the G1 phase, cells integrate the mitogenic as well as growth inhibitory signals, then, make decision to proceed, pause, or exit the cell cycle [11], [12]. There is growing evidence that the G1-S transition during cell cycle is also regulated by metabolic events, suggesting the possible existence of a metabolic or energetic checkpoint. Some studies have indicated that a round of cell cycle and mitochondrial oxidative capacity are greater at late G1 than early G1. Also, the reduction of mitochondrial ATP production blocks G1-S transition of the cell cycle, thus, affects mitochondrial function or shape [13], [14]. These studies have suggested that the metabolic checkpoint integrates not only external growth stimuli and nutrient availability but also the potential synchronization of intrinsic mitochondrial metabolism with cell cycle progression.

Leucine zipper/EF hand-containing transmembrane-1 (LETM1) is a mitochondrial inner membrane protein that was first identified in Wolf-Hirschhorn syndrome, and was deleted in nearly all patients with the syndrome [15]. LETM1 encodes for the human homologue of yeast Mdm38p, which is a mitochondria-shaping protein of unclear function. However, a previous study demonstrated that LETM1 served as an anchor protein for complex formation between mitochondria and ribosome, and regulated mitochondrial biogenesis [16]. Also, a recent study determined that LETM1 was associated with carboxyl-terminal modulator protein (CTMP), a negative regulator of Akt [17].

Together, the potential importance of LETM1 as a potential tumor suppressor gene with poor prognosis of diverse oncogenic nononcogenic lung diseases have prompted us to examine the possibility that LETM1 may function to regulate mitochondria and lung tumor growth. Here, we report that adenovirus-mediated LETM1 overexpression can induce the AMPK activity and inhibit the cell cycle progression through selective destruction of mitochondria with ATP depletion. Our results support the hypothesis that LETM1 may function as a tumor suppressor gene for lung cancer therapy as well as prevention.

## Results

### LEMT1 reduced mitochondrial ATP production and increased AMPK protein level

**Figure 1:**
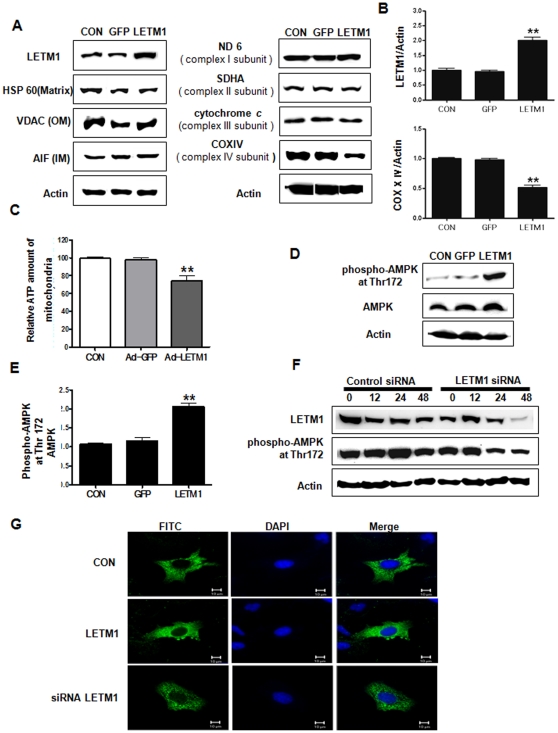
Effects of LETM1 on mitochondria biogenesis and AMPK activity. A549 cells were infected with GFP (Ad-GFP), LETM1 (Ad-LETM1), or without adenovirus (Control) for 48 h. (A) Mitochondria proteins were analyzed by immunoblotting with the indicated antibodies [mitochondrial-encoding respiration chain proteins: NADH: ubiquinone oxidoreductase 6 (ND6), a complex I subunit; succinate dehydrogenase complex subunit A (SDHA), a complex II subunit; and cytochrome *c*, complex III subunit; COXIV, a complex IV subunit and another mitochondrial protein: apoptosis-inducing factor (AIF), heat shock protein 60 (HSP60), and voltage-dependent anion channel (VDAC)]. (B) The bands-of-interest were further analyzed by densitometry. (C) Mitochondrial ATP was measured by luciferase assay. (D) Expression level of phospho-AMPK at Thr172 and AMPK proteins. (E) The bands-of-interest were further analyzed by densitometry.(F) A549 cells were transfected with siRNA LETM1 in time-dependent manner and then Western blot analysis of phospho-AMPK at Thr172. (G) Cells were fixed and immunostained with anti-phospho-AMPK (Thr172) antibody and then visualized on a confocal microscope. Each bars represent mean±SE (n = 3), *P<0.05 was considered significant and **P<0.01 highly significant compared with corresponding control values.

LETM1 plays a role in the regulation of mitochondrial network and biogenesis, thus, the expression levels of mitochondrial proteins were analyzed. Adenovirus-mediated LETM1 transfection increased the LETM1 protein while heat shock protein 60 (HSP60), the mitochondrial outer membrane (OM) protein voltage-dependent anion channel (VDAC), and the mitochondrial inner membrane (IM), and protein apoptosis-inducing factor (AIF) remained unchanged (Left columns of Fig. 1A and 1B). Among mitochondrial-encoding respiration chain proteins such NADH:ubiquinone oxidoreductase 6 (ND6), a complex I subunit; succinate dehydrogenase complex subunit A (SDHA), a complex II subunit; and cytochrome *c*, complex III subunit; cytochrome *c* oxidase IV (COXIV), a complex IV subunit, only the protein level of COXIV was selectively decreased (Right columns of Fig. 1A and 1B). To further evaluate the precise effects of a selective increase of LETM1 and decrease of COXIV, we monitored the change of ATP level. Analysis of relative mitochondrial ATP amounts using COX-8 luciferase revealed that the mitochondrial ATP level was significantly reduced in LETM1 overexpressed cells compared to control (Fig. 1C), indicating that LETM1 inhibited the production of mitochondrial ATP. Since AMPK is activated to reserve cellular energy content as described earlier, we investigated the changes in protein expression of total AMPK, phospho-AMPK at Thr 172 by LETM1 overexpression. Our result showed that LETM1 significantly increased phospho-AMPK at Thr 172 compared to total AMPK protein *in vitro* (Fig. 1D and 1E). Such AMPK activation has been reduced by the treatment of siRNA LETM1 as a function of time (Fig. 1F). Immnuofluorescence analysis also clearly confirmed that adenovirus-LETM1 increased the expression of phospho- AMPK at Thr 172 in A549 cells (Fig. 1G).

### LETM1 inhibited the Akt/mTOR pathway

**Figure 2:**
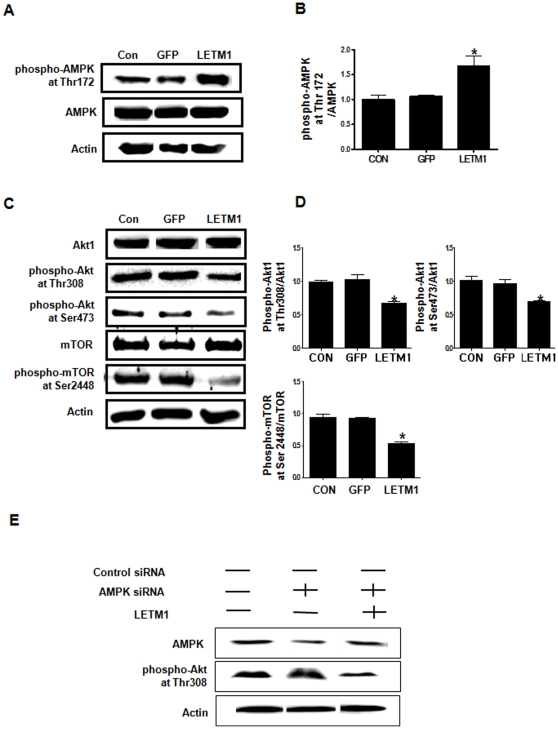
LETM1 inhibits Akt/mTOR pathway *in vivo*. The 9 weeks K*-ras* ^LA1^ mice were exposed to aerosols containing adenovirus-LETM1 twice a week for total 4 weeks. Lung tissue homogenates were subjected to western blot analysis. (A) Expression level of LETM1, phospho-AMPK at Thr172 and AMPK proteins. (B) The bands-of-interest were further analyzed by densitometry. (C) Expression level of Akt, phospho-Akt at Ser473, phospho-Akt at Thr308, mTOR and phospho-mTOR at Ser2448 proteins. (D) The bands-of-interest were further analyzed by densitometry. Each bar represents mean±SE (n = 8), *P<0.05 was considered significant and **P<0.01 highly significant compared with corresponding control values. (E) The effect of only siRNA AMPK or with infected adenovirus-LETM1 and protein expression level of AMPK and phospho-Akt at Thr 308.

The *in vitro* findings were also reproduced in K-*ras* ^LA1^ murine lung cancer model treated adenovirus-LETM1 via aerosol (Fig. 2A and 2B), suggesting that loss of ATP level in adenovirus-LETM1 infected cells causes AMPK activation. Energy depletion also affects the activity PI3-kinase/Akt pathway [18]. To investigate whether LETM1 overexpression would alter the Akt activity *in vivo*, Western blot analysis was performed. Our result showed that adenovirus-mediated LETM1 inhibited Akt1 activity significantly through suppression of phosphorylation at Thr308 and Ser473 as well (Fig. 2C). We also confirmed that knockdown of AMPK by siRNA and infected adenovirus-LETM1 inhibited Akt1 activity through suppression of phosphorylation at Thr308 (Fig. 2E). Also, adenovirus-mediated LETM1 significantly decreased phospho-mTOR at Ser 2448 expression level (Fig. 2C). Densitometry analysis of bands-of-interest clearly confirmed the result of Western blot analysis (Fig. 2D).

### LETM1 caused G1/S phase cell cycle arrest

**Figure 3:**
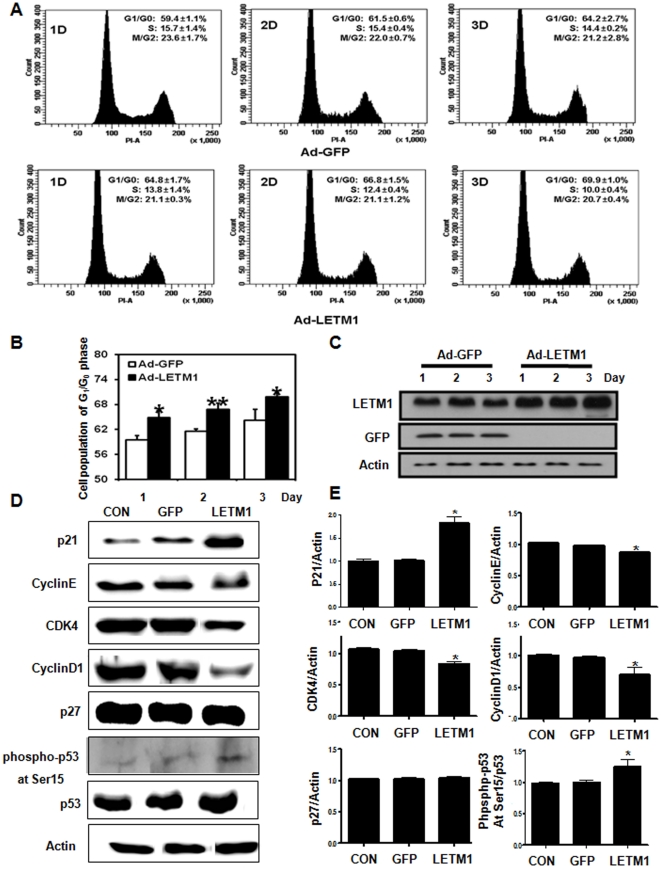
LETM1 causes cell cycle arrest in G1-S phase ***in vitro***. A549 cells was infected with GFP (Ad-GFP), LETM1 (Ad-LETM1), or without adenovirus (Control) for the indicated times. (A) Cell cycle distribution was determined by FACS. (B) Percentage of G1/G0 phase cell population. (C) Expression of LETM1 Protein for different times. (D) Expression of p21, cyclinE, cyclinD1, CDK4, p27, phospho-p53 at Ser15 and p53 proteins. (E)The bands-of-interest were further analyzed by densitometry. Each bars represent mean±SE (n = 3), *P<0.05 was considered significant and **P<0.01 highly significant compared with corresponding control values.

**Figure 4:**
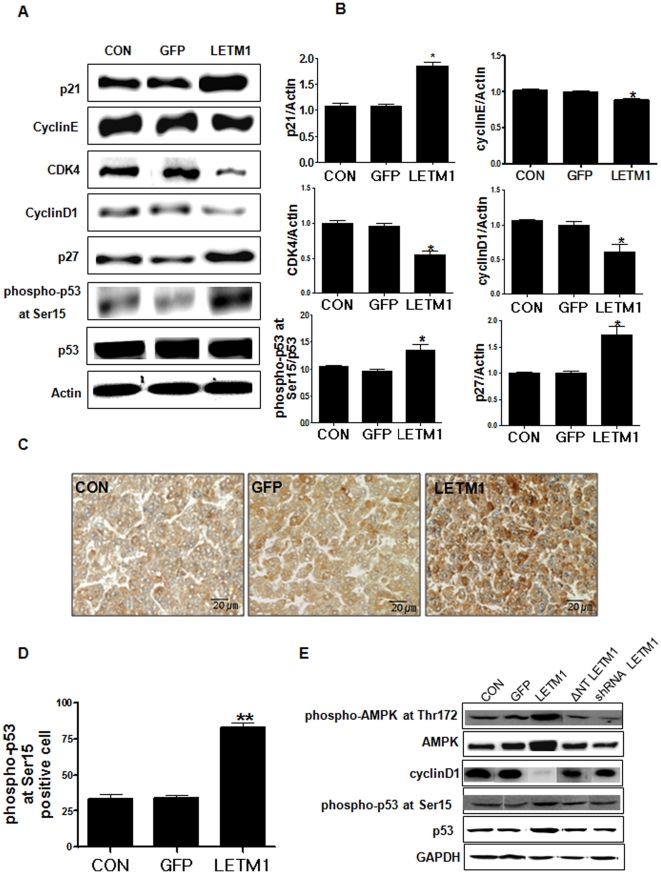
LETM1 induced cell cycle arrest at the G1/S phases ***in vitro***. The 9 weeks K*-ras* ^LA1^ mice were exposed to aerosols containing adenovirus-LETM1 twice a week for total 4 weeks. Lung tissue homogenates were subjected to western blot analysis. (A) Expression of p21, cyclinE, cyclinD1, CDK4, p27, phospho-p53 at Ser15 and p53 proteins. (B) The bands-of-interest were further analyzed by densitometry (n = 8). (C) Immunohistochemistry analysis of phospho-p53 at Ser 15. Dark brown color indicates the expression (Scale bar = 20 µm). (D) Comparison of phospho-p53 at Ser15 labeling index. The phospho-p53 at Ser15 positive staining was determined by counting 10 randomly chosen fields per section, determining the percentage of DAB positive cell per 100 cells. Each bar represents mean±SE (n = 8), *P<0.05 was considered significant and **P<0.01 highly significant compared with corresponding control values. (E) Reversal of LETM1-mediated events by MTS-deleted LETM1-adenovirus and shRNA-LETM1. Expression of phospho-Akt1 at Thr308, phospho-Akt1 at Ser473, Akt1, phospho-AMPK at Thr172, AMPK, cyclinD1, phospho-p53 at Ser15 and p53 proteins.

Since AMPK pathway functions as a cellular energy-sensing checkpoint that controls cell proliferation [19], the effects of LETM1 on cell proliferation were measured. Analysis of cell cycle distribution using flow cytometry revealed that LETM1 significantly increased the G1/G0 subpopulation in time-dependent manner (Fig. 3A and 3B), suggesting that LETM1 inhibited cell cycle progression from G0/G1 into S phase as a function of LETM1 overexpression (Fig. 3C). To further confirm these results, several representative cell cycle-related proteins were analyzed by Western blot analysis. As shown in Fig. 3D and E, adenovirus-mediated LETM1 suppressed the proteins important for cell cycle regulation such as cyclin D1, cyclin-dependent kinase 4(CDK4) in A549 cells. In contrast, cellular levels of p53 phosphorylation and inhibitors of CDK such as p21 were increased significantly (Fig. 3D and 3E). Such changes of cell cycle-related genes were also found similarly in *in vivo* study. As shown in Figure 4A and B, aerosol delivery of adenovirus-mediated LETM1 suppressed the protein expression of CDK4 and cyclin D1, however, the level of p21, p27 and phospho-p53 at Ser15 were increased. Increased p53 phosphorylation at Ser15 was further confirmed by immunohistochemistry (Fig. 4C) and statistical analysis of phospho-p53 at Ser15 positive cell counts (Fig. 4D) in the lung of K-*ras* ^LA1^ mice treated with aerosol-containing adenovirus-LETM1. We also confirmed that all changes including AMPK activation as well as cell cycle arrest were closely associated with LETM1 because LETM1 knockdown (shRNA LETM1) as well as the blocking of LETM1 delivery into mitochondria reversed the LETM1-mediated events (Fig. 4E).

### LETM1 altered mitochondrial morphology

**Figure 5:**
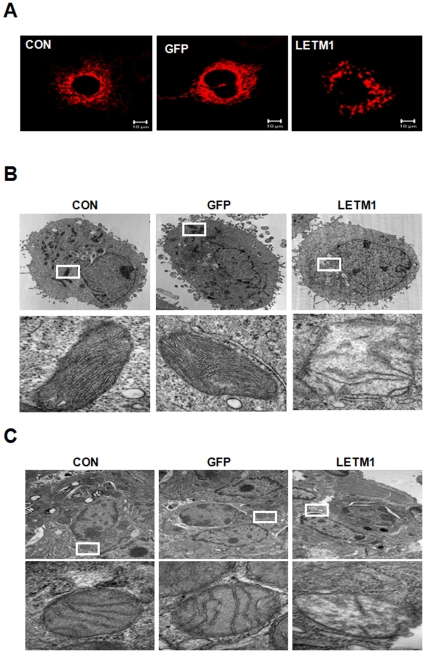
LETM1 induced mitochondrial dysmorphology. (A) A549 cells that stably expressed mitochondrion-targeted Mito-DsRed were infected with GFP (Ad-GFP), LETM1-adenovirus (Ad-LETM1) or without adenovirus (Control) for 48 h and imaged via confocal microscopy. (B) Mitochondrial ultrastructure was analyzed by transmission electron microscopy in A549 cells. (C) Mitochondrial ultrastructure was analyzed by transmission electron microscopy in the lungs of 9 weeks K*-ras* ^LA1^ mice. Bottom panels are the magnification of the indicated area (Scale bar = 100 nm).

Mitochondria are morphologically dynamic organelles that continuously divide and fuse to form small individual units or interconnected networks within the cell. To detect the mitochondrial morphologic change after overexpressing LETM1, analysis by confocal laser scanning microscopy (CLSM) and transition electron microscopy (TEM) was performed. Overexpression of LETM1 in A549 cells caused fragmented pattern of mitochondria morphology demonstrated by CLSM (Fig. 5A). TEM analysis clearly demonstrated the disruption of mitochondria cristae organization in A549 cells (Fig. 5B). Such destruction of mitochondrial cristae was clearly reproduced in the lung of K-*ras* ^LA1^ mice treated with aerosol-containing LETM1 (Fig. 5C). These results demonstrate clearly that LETM1 causes mitochondrial morphologic changes.

### LETM1 induced *in vitro* and *in vivo* apoptosis

**Figure 6:**
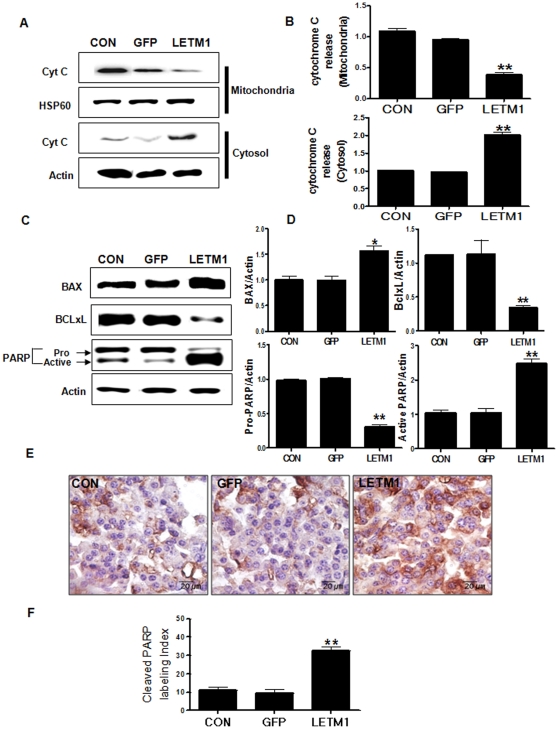
LETM1 induced apoptosis. A549 cells was infected with GFP (Ad-GFP), LETM1 (Ad-LETM1), or without adenovirus (Control). (A) Western blot analysis of the release of cytochrome *c* from mitochondria. (B) The bands-of-interest were further analyzed by densitometry. Each bar represents mean±SE (n = 3), *P<0.05 was considered significant and **P<0.01 highly significant compared with corresponding control values. The 9 weeks K*-ras* ^LA1^ mice were exposed to aerosols containing adenovirus-LETM1 twice a week for total 4 weeks. Lung tissue homogenates were subjected to Western blot analysis. (C) Expression level of BAX, BclxL, PARP proteins. (D) The bands-of-interest were further analyzed by diameter. (E) Immunohistochemical analysis of cleaved PARP. Dark brown color indicates the expression (Scale bar = 20 µm). (F) Comparison of cleaved PARP labeling index. Cleaved PARP positive staining was determined by counting 10 randomly chosen fields per section, determining the percentage of DAB positive cell per 50 cells. Each bar represents mean±SE (n = 8), *P<0.05 was considered significant and **P<0.01 highly significant compared with corresponding control values.

It is known that the anti-apoptotic Bcl-2 family members, such as Bcl-2 and Bcl-XL, are able to bind BAX and sequester them in protein complexes to prevent triggering of apoptosis [20]. However, activation of BAX has been proposed to result in formation of homomultimeric pore, permeabilization of mitochondrial membrane to initiate cytochrome *c* release, suggesting a mechanistic link between mitochondrial morphology and apoptosis. Our results demonstrated that overexpression of LETM1 resulted in release of cytochrome *c* from the mitochondria to cytosol (Fig. 6A and 6B). Also, adenovirus-LETM1 reduced mitochondrial membrane potential compared with GFP and Control group (Fig. S1A). Futhermore, annexin V flow cytometric analysis reconfirmed the above findings. As shown in Fig. S1C, 28.20% of Annexin V-positive cells were detected in A549 cells infected with adenovirus-LETM1 compared to control and others. To further confirm this finding in *in vivo* murine experiment, we delivered adenovirus-LETM1 to *K-ras* ^LA1^ mice via aerosol and examined the changes in protein expression of apoptosis-related proteins. Our result showed that aerosol delivery of LETM1 significantly increased BAX and active-PARP protein level while decreasing the Pro-PARP protein. In contrast, aerosol delivered-LETM1 decreased Bcl-xL protein level in the lung of LETM1-delivered mice (Fig. 6C, 6D and Fig. S2). Immunohistochemistry analysis also clearly confirmed that aerosol delivered adenovirus-LETM1 increased the expression of active-PARP in the lung of K-*ras* ^LA1^ mice (Fig. 6E and 6F). These results clearly indicated that adenovirus-LETM1 induced apoptosis both *in vitro* and *in vivo*.

### LETM1 inhibited lung tumorigenesis

**Figure 7:**
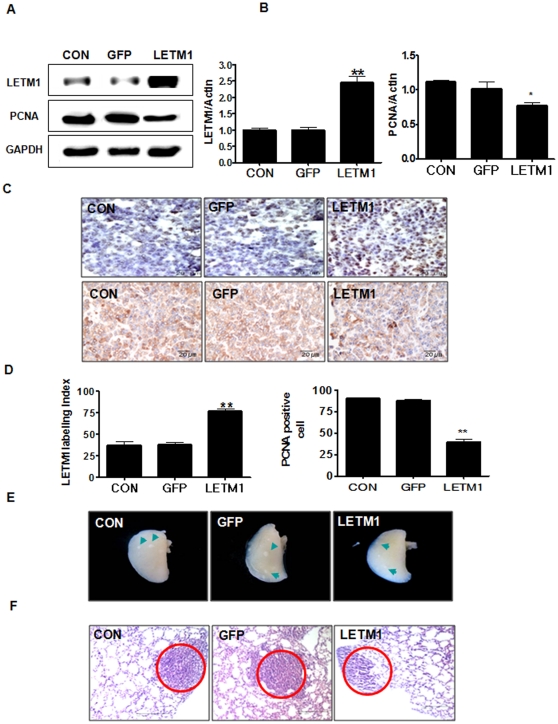
LETM1 suppressed tumor growth. The 9 weeks K*-ras* ^LA1^ mice were exposed to aerosols containing adenovirus-LETM1 twice a week for total 4 weeks. Lung tissue homogenates were subjected to western blot analysis. (A) Expression of LETM1 and PCNA proteins. (B) The bands-of-interest were further analyzed by densitometry. (C) Immunohistochemistry analysis of LETM1 (upper panel; Scale bar = 20 µm) and PCNA (lower panel; Scale bar = 20 µm). Dark brown color indicates the expression. (D) Comparison of LETM1 (left) and PCNA (right) labeling index. LETM1 and PCNA positive staining was determined by counting 10 randomly chosen fields per section, determining the percentage of DAB positive cell per 100 cells. Each bar represents mean±SE (n = 8). *P<0.05 was considered significant and **P<0.01 highly significant compared with corresponding control values. (E) K*-ras* ^LA1^ mice lungs with whitish multiple nodules in the lung (green arrows). (F) Histological examination of K*-ras* ^LA1^ mice lungs (Scale bar  = 100 µm).

To investigate the effect of adenovirus-LETM1 on the growth of lung cancer, we examined such potential anti-tumor effects in *K-ras* ^LA1^ mice. For the purpose, we analyzed the effect of LETM1 on tumor cell proliferation using proliferating cell nuclear antigen (PCNA) antibody. LETM1 significantly reduced the expression and number of cells exhibiting PCNA expression (Fig. 7A and 7B). Immunohistochemistry and PCNA positive cell labeling index analysis demonstrated that aerosol delivered adenovirus-LETM1 decreased the expression of PCNA in the lung of K-*ra*s ^LA1^ mice significantly (Fig. 7C and 7D). Our results clearly indicated that adenovirus-LETM1 suppressed cancer cell proliferation in the lungs of human lung cancer model mice (Fig. S2A and S2B). Also, similar results were also observed in cell proliferation assays (Fig. S1B). Furthermore, histopathological analysis was performed in the lungs of *K-ras* ^LA1^ mice. Aerosol delivery of LETM1 twice a week for a month suppressed lung tumor mass (Fig. 7E, green arrows). Such suppression of lung tumor formation was further confirmed by histopathological examination (Fig. 7F, red circles). The nodules developed in the lungs of control group (CON) and GFP-treated group (GFP) were in general alveolar/bronchial adenoma. In contrast, the lung hyperplasia was frequently observed in LETM1-treated group (LETM1) in which progression to adenoma was inhibited as shown in Fig. 7F and Table 1. Furthermore, the total tumor number was significantly decreased by adenoviral LETM1 delivery in the lungs. Also, tumor angiogenesis is an important prognostic marker for many solid tumors, including lung cancer that independently predicts pathologic stages of tumor progression and tumor growth rate [21]. Therefore, we examined expression level of MMP-2, MMP-9, CD31 and VEGF in the lungs of K-*ras* ^LA1^ mice. Aerosol delivery of LETM1 decreased MMP-2 and MMP-9 protein level compared to those of control and vector control (Fig. S3). These results showed that LEM1 overexpression may play a key role in suppressing the lung cancer growth and progression (summery in table1, Fig 7 and Fig. S4).

**Table 1:** Summary of tumor incidence in the lungs of K-*ras* ^LA1^ mice.

Group	NO	Tumor number	Adenoma	Hyperplasia
CON	8	10.6±2.26	A(5)^++^	B(3)^+++^
GFP	8	10.7±3.27	A(4)^+++^	B(4)^+++^
LETM1	8	6.37±1.99**	A(2)^+^	B(6)^++^

A, alveolar/bronchiolar adenoma; B, alveolar epithelial cell hyperplasia.

No, number of K-*ras* ^LA1^ mice per group.

CON, control; GFP, adenovirus-GFP treated group; LETM1, adenovirus-LETM1 treated group.

( ), number of mice.

Grade: +, minimal; ++, mild; +++, severe.

**, denote statistical different (**, p<0.01) compared to control.

## Discussion

Mitochondria are key players in several cellular functions including growth, division, energy metabolism, and apoptosis [22]. Mitochondrial dysfunctions have been associated with various human diseases including cancers [23].

Recent findings have shown that LETM1 regulates mitochondrial biogenesis and translation system which can be implicated in tumorigenesis and cancer [17]. Therefore, this study was designed to investigate the tumor suppressor function of LETM1 and clarify the mechanism underlying tumor growth inhibition in lung cancer model.

It has been shown that functional mitochondria are required for all cell processes due to common energetic requirements [24]. Cytochrome *c* oxidase or complex IV, catalyzes the final step in mitochondrial electron transfer chain, and is regarded as one of the major regulation sites for oxidative phosphorylation. A detail biosynthetic and functional analysis of several cell lines with suppressed COX IV expression revealed a loss of assembly of cytochrome *c* oxidase complex and, correspondingly, a reduction in cytochrome *c* oxidase-dependent respiration and total respiration [25]. Furthermore, dysfunctional cytochrome *c* oxidase in the cells leads to a decreased ATP level. In our study, we showed that LETM1 overexpression decreased specific mitochondrial protein cytochrome *c* oxidase subunit IV resulting in a reduction of mitochondrial ATP level in lung cancer cells (Fig. 1). AMPK is now emerging as a modulator of different cellular responses, all converging in the restoration of ATP levels. Elevated AMP/ATP ratio activates AMPK, which inhibits energy-consuming processes and activates energy-producing processes to restore the energy homeostasis in the cell [26]. In this study, increased LETM1 phosphorylates AMPK on Threonine172 leading to activation of the energy sensor AMPK (Fig. 1D, 1E, 1F, Fig. 2A, 2B, 2E and Fig. 4E). Our findings are supported by recent works that AMPK activation led to inhibition of prostate cancer cell growth [10] and anti-pulmonary tumor effects of deguelin by AMPK activation with Akt inactivation [27].

Akt controls a number of metabolic and survival pathway and was recently found to translocate into the mitochondria matrix in response to insulin stimulation. mTOR, as a downstream protein of Akt, is a key regulator of protein synthesis responding to growth factor as well as metabolic control [28]. AMPK has been reported to inhibit the activity of mTOR and the phosphorylation-mediated activation of 70-kDa ribosomal protein S6 kinase (p70S6K), events linked to the inhibition of cell proliferation and induction of a pro-apoptotic environment [29]. Our results clearly showed that aerosol delivery of LETM1 decreased Akt phosphorylation at Thr308/Ser473 and mTOR phosphorylation at Ser2448, respectively. However, an increase was observed in the levels of phosphorylated AMPK (Fig. 2C and 2D). These results strongly suggest that AMPK activation by overexpression of LETM1 can alter cancer cell growth through the inhibition of Akt/mTOR signaling pathway.

Recent studies have suggested that inhibitors of CDK activity are important mediators of observed cell cycle arrest and appear to act downstream of AMPK activation and cyclin D1 loss. Other AMPK activators such as AICAR and antimycin A also caused downregulation of cyclin D1. Downregulation of cyclin D1 may lead to release of p27 and p21, allowing them to bind to CDK2 and block its activity [30]. Our results also observed the decrease of cyclinD1, cyclin E and increase of p21, suggesting that activated AMPK by LETM1 inhibits G1/S cell cycle progression (Fig. 3 and Fig. 4). Recently, activated AMPK was shown to phosphorylate at Ser15 of p53, a modification that is known to protect the protein from degradation and promote cell cycle arrest during DNA damage [31]. In our study, activated AMPK by LETM1 led to a significant increase in p53 phosphorylation on Sernine15 resulting in cell cycle arrest (Fig. 3D and Fig. 4E).

We also found that overexpression of LEMT1 promoted apoptosis of lung cancer cells. The underlying mechanisms of these effects can relate to the role of LETM1 in the regulation of mitochondrial functions. Mitochondria are known to take active part in the apoptotic process by various mechanisms including energy metabolism, disruption of electron transport. The mitochondria apoptosis pathway is signified by the release of cytochrome *c*, a change in the mitochondria morphology and permeability. Bax interacts with intrinsic mitochondria proteins such as voltage-dependent anion channel to induce cytochrome *c* release from mitochondria [32]. Similarly, our studies found that overexpression of LETM1 induced Bax as well as cytochrome *c* release from mitochondria (Fig. 6). We also detected fragmented mitochondria and disorganization of mitochondrial cristae in adenovirus LETM1 infected cells as well as murine lung (Fig. 5), suggesting that LETM1 may play an important role in mitochondrial apoptosis.

Taken together these results demonstrated that such activation of AMPK activity led to cell cycle arrest and induction of apoptosis closely associated with LETM1 overexpression may play a key role in suppressing the lung cancer growth and progression (summery in table1 and Figure S4). In conclusion, our data suggest that LETM1 suppressed lung tumor growth through activation of AMPK activity and inhibition of Akt activity. Therefore, understanding the role of LETM1 may be important in developing effective therapeutics for lung cancer. Also, our current results in combination of detailed new information of the mechanism of AMPK action would provide us a good rationale to take LETM1 as a candidate for cancer therapy.

## Materials and Methods

### Antibodies and Reagents

Monoclonal antibodies against Akt1 and phospho-Akt at Ser473 were generated via a general method described elsewhere [33]. Anti-LETM1 antibody was purchased from Novus Biological (Littleton, CO, USA). Anti-COX IV, anti-VDAC, anti-Hsp60, anti-SDHA, anti-AIF and anti-ND 6 antibodies were purchased from Abcam (Cambridge, MA, USA). Anti-phospho-Akt at Thr308, anti-Bax, anti-BclxL, anti-cyclinE, anti-cyclinD1, anti-cyclinE, anti-p27, anti-p21, anti-CDK4, anti-AMPK, anti-cytochrome *c* anti-CD31, anti-VEGF, anti-MMP-2, anti-MMP-9 and anti-Actin antibodies were from Santa Cruz Biotechnology (Santa Cruz, CA, USA). Anti-cleaved PARP, anti-phospho-p53 at Ser15, anti-p53, anti-phospho-AMPK at Thr172, anti-mTOR, and anti-phospho-mTOR at Ser2448 antibodies were obtained from Cell Signaling (Danvers, MA, USA). HRP conjugated anti-mouse and anti-rabbit IgG antibodies were from Zymed (San Francisco, CA, USA).

### Cell culture, Adenovirus infection

Human non-small cell lung cancer cell A549 was purchased from American Tissue Type Culture Collection (ATCC, Manassas, VA, USA). The cells were maintained in RPMI 1640 (Invitrogen, Carlsbad, CA, USA) with 10% heat-inactivated fetal calf serum (FCS), 100,000 units/L penicillin, and 100 mg/L streptomycin (Invitrogen) at 37°C in 5% CO_2_ incubator. Stable cell lines expressing pDsRed-Mito were isolated after selecting for 3 weeks with G418 (Sigma-Aldrich, St. Louis, MO, USA). Adenoviral expression vector for wild-type LETM1 (LETM1), GFP, shRNA LETM1 and MTS-deleted LETM1 were prepared by using Adenoviral Expression Kit (Invitrogen). A549 cells were infected with the appropriate adenovirus-LETM1, adenovirus-GFP, adenovirus-shRNA LETM1 and adenovirus- MTS-deleted LETM1 (10^4^ PFU/ml) for the indicated time when cells reached 80% confluence. The medium was changed 6 h post-infection, and incubation for 48 h.

### Flow cytometrical analysis of cell cycle

Cells were cultured in 6-well plates and infected with adenovirus-LETM1, adenovirus-GFP for designated time points. Cell attached to the plate were collected with trypsin, washed, and resuspended in 100 µl of PBS, and 5 ml of 70% ethanol was added slowly while continuous vortexing of cells and were fixed overnight. Next day, cell were spun, washed, and suspended in 400 µl of PBS with addition of 10 mg/L RNase A and 75 µM propidium iodide and analyzed by FACS Calibur (BD Bioscience, San Jose, CA, USA).

### Small interference RNA (siRNA) and small hairpin RNA (shRNA) transfection

To knock down the endogenous LETM1, A549 cells were transiently transfected with 10 nM of the chemically synthesized siRNAs/shRNAs targeting AMPK and/or LETM1 or with the nonsilencing control siRNA/shRNA using Transfection Reagent according to the manufactor's recommendations (Santa Cruz Biotechnology). Whole cell lysates were prepared 48 h after transfection. siRNA/shRNA sequences used in the present study are available upon request.

### Measurement of mitochondria ATP

Wild-type firefly luciferase from pGL3-base vector was subcloned into pcDNA3.1-HA vector. For the mitochondrial luciferase (Luc-m), it was fused downstream of the sequence encoding the mitochondrial targeting signal (MTS) of subunit 8 of cytochrome *c* oxidase. The COX 8 targeting sequence has been used successfully in other mitochondrial targeting experiments, and a similar COX 8-luciferase construct was shown to be targeted appropriately to the mitochondrial matrix [34]. Light emission was measured in a luminometer at 5-S intervals until the maxium value of luminescence was reached. To normalize for the variability of luciferase expression in transfected cells, the relative luminescence values in each cell compartment were expressed as a ratio to the total potential luminescence measured on equal aliquots of the same lysed cells with a luciferase assay kit (Promega, Madison, USA) in the presence of excess ATP.

### Transmission electron microscopy (TEM)

A549 cells and lung cancer tissue were fixed with a solution of 2.5% glutaraldehyde with 1% Osimium tetroxide (OsO_4_) buffer for 2 h at 4°C and dehydrated with ethanol at 4°C. Then cells and tissues were infiltrated in a 1∶1 mixture of propylene oxide and Epon and finally embedded in Epon by polymerization at 70°C for 24 h. Ultrathin sections (40–70 nm) were cut and mounted on pioloform-coated copper grids. Sections were stained with lead citrate and uranyl acetate and viewed with a JEM 1010 transmission electron microscope (JEOL, Tokyo, Japan).

### Confocal imaging analysis and indirect immnuofluorescence

Mitochondria expression stable cells (pDsRed-Mito) were grown 2-well chamber slides until 50–70% confluent and then infected with adenovirus-GFP or adenovirus-LETM1 for 6 h. After 48 h, the cells were fixed with 4% paraformaldehyde at room temperature for 10 min, mount with Dakocytomation Faramount Aqueous Mounting solution (Dakocytomation, carpinteria, CA, USA) and visualized using a Carl Zeiss confocal microscope (Carl Zeiss MicroImaging, Thronwood, NY, USA). For indirect immunofluorescence, A549 cells were fixed with 4% paraformaldehyde at room temperature for 10 min and permeabilized with 1% Triton X-100 at room temperature for 15 min. Then cells were incubated in blocking buffer containing 3% bovine serum albumin (Sigma) in 1× TBS for 1 h at 37°C. The mouse monoclonal anti-phospho-AMPK (Thr172) was diluted 200 fold for primary antibody and incubated for overnight. The secondary antibody, FITC-conjugated anti-mouse antibody (BD Biosciences) was used. After appropriate rinsing, coverslips were mounted in Dakocytomation Faramount Aqueous Mounting solution on glass slides and analyzed.

### Isolation of mitochondria and cytosolic protein

A549 cells were washed with PBS and resuspended in mitochondrial fraction buffer (20 mM HEPES, pH 8.0, 10 mM KCl, 1.5 mM MgCl_2_, 1 mM EDTA, 250 mM sucrose, 1 mM PMSF, 10 g/ml leupeptin, 10 g/ml aprotinin, and 0.2 mM sodium orthovanadate) for 30 min on ice, and then homogenized. Unbroken cells and nuclei were pelleted by centrifugation at 1500 g for 10 min. The supernatant was continuously centrifuged at 10,000 g for 30 min at 4°C and transferred to a new tube for the post-mitochondrial fraction. Next, the supernatant was centrifuged at 100,000 g for 1 h at 4°C, and the supernatant was used as a cytosolic fraction. The post-mitochondrial fraction pellet was washed with 500 µl of mitochondrial fraction buffer, and used for isolation of the mitochondrial fraction. The supernatant and mitochondrial fractions were collected and protein concentration was determined by the Bradford assay. Equivalent amounts of protein were loaded and electrophoresed on SDS–polyacrylamide gels. The membrane was incubated with the following primary antibodies: mouse anti-cytochrome c (Santa Cruz) and rabbit anti-HSP60 (Abcam, Cambridge, MA, USA) followed by incubation with ahorseradish peroxidase conjugated secondary antibodies.

### *In vivo* aerosol delivery of adenovirus-LETM1

Experiments were carried out on 9-week-male K*-ras* ^LA1^ mice (8mice/group). The breeding mice were obtained from Human Cancer Consortium-National Cancer Institute (Frederick, MD, USA) and kept in the laboratory animal facility with temperature and relative humidity maintained at 23±2°C, with a and 50±20% respectively and were kept on a 12- hour light/dark cycle. All methods used in this study were approved by the Animal Care and Use Committee at Seoul National University (SNU-071210-7). For gene delivery, mice were placed in nose-only exposure chamber and exposed to the aerosol based on the methods used previously. Our aerosol gene delivery methods have been successfully applied in diverse studies [33], [35], [36], [37], [38]. For the effects of LETM1 against lung cancer development, the K*-ras* ^LA1^ mice were divided into three groups. Control group was not treated with anything and other two groups were exposed to aerosol containing adenovirus-GFP or adenovirus-LETM1 (5×10^6^ PFU/ml). The K*-ras* ^LA1^ mice were exposed to aerosols twice a week for total 4 weeks. At the end of the study, K*-ras* ^LA1^ mice were sacrificied, and the lungs were collected. During the autopsy procedure, the neoplastic lesions of lung surfaces were carefully counted and the lesion diameter was measured with the aid of digital calipers under microscope as described [39]. Simultaneously, the lungs were perfused and fixed in 10% neutral-buffered formalin for histophathological studies.

### Histopathological analysis and immunohistochemistry (IHC)

The lung tissues were fixed in 10% neutral buffered formalin-fixed, paraffin-embedded tissue section were cut and transferred to plus slides (Fisher Scientific, Pittsburgh, PA, USA). For histological analysis, the tissue sections were stained with hematoxylin and eosin (H&E). For IHC, the tissue section were deparaffinized in xylene and rehydrated through alcohol gradients, then washed and incubated in 3% hydrogen peroxide (AppliChem, Boca Raton, FL, USA) for 30 min to quench endogenous peroxidase activity. After washing in PBS, the tissue sections were incubated with 3% BSA in PBS for 1 h at room temperature to block the unspecific binding sites. Primary antibodies were applied on tissue section for overnight. The following day, the tissue sections were washed and incubated with secondary HRP-conjugated antibodies (1∶50; Zymed) for 2 hr at room temperature. After washing, tissue sections were counterstained with Mayer′s Hematoxylin (DAKO, Carpinteria, CA, USA) and washed with xylene. Cover slips were mounted using Permount (Fisher Scientific), and the slides were reviewed using alight microscope (Carl Zeiss). Staining intensity of phospho-p53 at Ser15, LETM1, PCNA, ki-67, BCLxL and cleaved PARP for quantification of IHC analysis were performed using In Studio version 3.01 programs (Pixera, San Jose, CA, USA). Staining intensity was assessed by counting the number of positive cells in randomly selected fields viewed with appropriate magnification of objective lens.

### Western Blot Analysis

Cells and tissue were placed on ice and extracted with lysis buffer containing 50 mM Tris-HCl, pH 7.5, 1% v/v Nonidet P-40, 120 mM NaCl, 25 mM sodium fluoride, 40 mM glycerol phosphate, 0.1 mM sodium orthovanadate, 1 mM phenylmethylsulfonyl fluoride, 1 mM benzamidine, and 2 µM microcystin-LR. Lysates were centrifuged for 15 min at 12,000 g. Cell extracts were resolved by 12% SDS-PAGE and transferred to nitrocellulose membranes (Amersham Pharmacia, Pittsburgh, PA, USA). The membranes were blocked for 1 hr in TTBS (Tris-buffered saline+Tween 20) containing 5% skim milk, and immunoblotting was done by incubating overnight, and then with secondary antibodies conjugated to horseradish peroxides (HRP) for 3 hours at room temperature or overnight. After washing, the bands-of-interests were pictured by luminescent image analyzer LAS-3000 (Fujifilm, Tokyo, Japan), and quantification of Western blot analysis was done by using multi Gauge version 2.02 program (Fujifilm).

### Mitochondrial membrane potential analysis

To analyze the mitochondria membrane potential, A549 cells were cultured in 2well-chamber slide and infected with adenovirus-LETM1 (LETM1), adenovirus-GFP (GFP) and for 6 h. After 48 h, cell were incubated 20 min with JC-1 (5,5′,6′,6′-tetrachloro-1,1′,3,3′-tetaethylbenzimi-dazolylcarbocyanine iodide, Molecular Probes) in culture medium. The dye concentrates in mitochondria maintaining a high membrane potential and forms aggregates having a red emission spectrum. The monomeric form of JC-1 has a green emission spectrum. After rinsing the slides, the red and green emissions of JC-1 were detected with the TRITC and FITC photomultipliers of the confocal microscope, respectively. Fluorescence images visualized using a Carl Zeiss confocal microscope (Carl Zeiss MicroImaging).

### Flow cytometrical analysis of apoptosis

Cells were cultured in T-75 Flask and infected with adenovirus-LETM1, adenovirus-GFP for 6 h. After 48 h, cell were collected with trypsin, washed, and resuspended in 1× Binding Buffer at a concentration of 1×10^6^/ml and 100 µl of the solution (5×10^5^ cells) were transferred in a 5 ml FACS tube and combined with 1 µl Annexin V/FITC and 1 µl propidium iodide. After incubation for 30 min at room temperature in the dark, 300 µl of 1× Binding Buffer were added to each tube. The rate apoptosis was measured using the Annexin V/FITC apoptosis detection kit (Abcam) with FACS Calibur (BD Bioscience).

### Gelatin zymography assay

The tumor tissues were homogenized and protein was measured using the Bradford kit (Bio-Rad). Equal amounts of protein were electrophoresed in 10% SDS-polyacrylamide gel containing 1% gelatin. The gel was then washed at room temperature for 2 h with 2.5% Triton X-100 and subsequently incubated at 37°C in a buffer containing 10mmol/L CaCl2, 150mmol/L NaCl, and 50mmol/L Tris–HCl (pH 7.5) for 48 h. The gel was then stained with Coomassie blue to visualize gelatinolytic activity.

### Statistical Analysis

Data are expressed as the mean ± S.E. from at least three separate experiments performed triplicate. The differences between groups were analyzed using a Student's *t* test (Graphpad Software, San Diego, CA, USA). p<0.05 (*) was considered significant, and p<0.01 (**) was highly significant compared with corresponding control values. Quantification of western blot analysis was done by using Multi Gauge Version 2.02 program (Fujifilm).

## Supporting Information

Figure S1 LETM1 decreased mitochondrial membrane potential and induced apoptosis in A549 cells. (A) Confocal pictures of cells stained with JC-1 dye. The JC-1 red to green ratio was used to monitor mitochondria membrane potential. The red fluorescence of JC-1 monomers indicates high mitochondria membrane potential while green fluorescence of JC-1 monomers indicates mitochondria with depolarized membranes. (B) Cell proliferation assay. (C) Apoptotic cell-death was measured using the Annexin V/FITC apoptosis detection kit (Abcam) with FACS Calibur (BD Bioscience). Quatitative measurement of Annexin V/FITC flow cytometry analyses showing positive apoptotic cells.
(0.69 MB TIF)

Figure S2 Effect of aerosol-delivered LETM on lung tumor angiogenesis. (A) Gelatin zymography assay and western blot analysis for activity of matrix metalloproteinase-2 (MMP-2). (B) The bands-of-interest were further analyzed by diameter. (C) Western blot analysis of VEGF, CD31and MMP-9 proteins in the lungs of K-*ras* ^LA1^ mice. (D)The bands-of-interest were further analyzed by diameter.
(1.38 MB TIF)

Figure S3 Immunohistochemistry of ki-67 and BCLxL in the lungs of K-*ras* ^LA1^ mice. (A) Immunohistochemistry analysis of ki-67 in the lung. Dark brown color indicates the Ki-67 expression (magnification, X 100 and X400). (B) Comparison of ki-67 labeling index. ki-67 positive staining was determined by counting 10 randomly chosen fields per section, determining the percentage of DAB positive cell per 50 cells. (C) Immunohistochemistry analysis of BCLxL in the lung. Dark brown color indicates the BCLxL expression (magnification, X 100 and X400). (D) Comparison of BCLxL labeling index. BCLxL positive staining was determined by counting 10 randomly chosen fields per section, determining the percentage of DAB positive cell per 50 cells. Each bar represents mean±SE (n = 8), *P<0.05 was considered significant and **P<0.01 highly significant compared with corresponding control values.
(0.42 MB TIF)

Figure S4 Schematic signal pathway by which LETM1 regulates lung cancer cell growth. LETM1 induced the activation of AMPK. Such activated AMPK inhibited Akt/mTOR signaling pathway and cyclinD1 expression level then decreased p53 and p21 expression level resulting in induction of apoptosis.
(0.32 MB TIF)
