# Supplementary figures and images for: Suppression of Lung Tumorigenesis by Leucine Zipper/EF Hand–Containing Transmembrane-1
Source: PLoS One. 2010 Sep 2;5(9):e12535. doi: 10.1371/journal.pone.0012535 (PMC2932724; doi:10.1371/journal.pone.0012535)

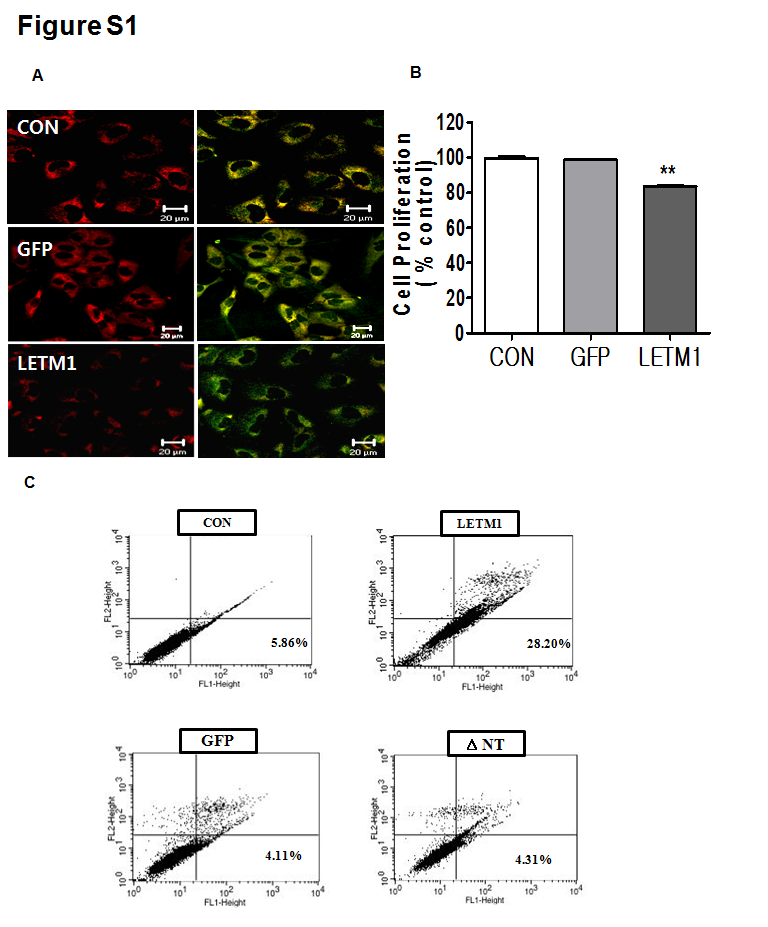

Supplement: Figure S1 — LETM1 decreased mitochondrial membrane potential and induced apoptosis in A549 cells. (A) Confocal pictures of cells stained with JC-1 dye. The JC-1 red to green ratio was used to monitor mitochondria membrane potential. The red fluorescence of JC-1 monomers indicates high mitochondria membrane potential while green fluorescence of JC-1 monomers indicates mitochondria with depolarized membranes. (B) Cell proliferation assay. (C) Apoptotic cell-death was measured using the Annexin V/FITC apoptosis detection kit (Abcam) with FACS Calibur (BD Bioscience). Quatitative measurement of Annexin V/FITC flow cytometry analyses showing positive apoptotic cells. (0.69 MB TIF) [file pone.0012535.s001.tif]

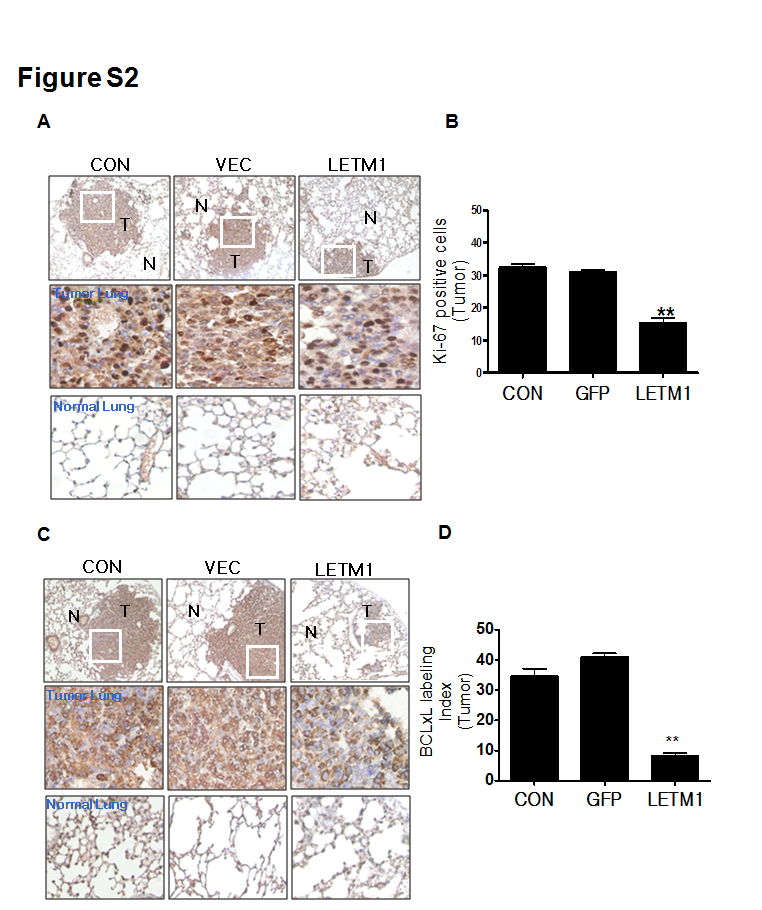

Supplement: Figure S2 — Effect of aerosol-delivered LETM on lung tumor angiogenesis. (A) Gelatin zymography assay and western blot analysis for activity of matrix metalloproteinase-2 (MMP-2). (B) The bands-of-interest were further analyzed by diameter. (C) Western blot analysis of VEGF, CD31and MMP-9 proteins in the lungs of K-ras LA1 mice. (D)The bands-of-interest were further analyzed by diameter. (1.38 MB TIF) [file pone.0012535.s002.tif]

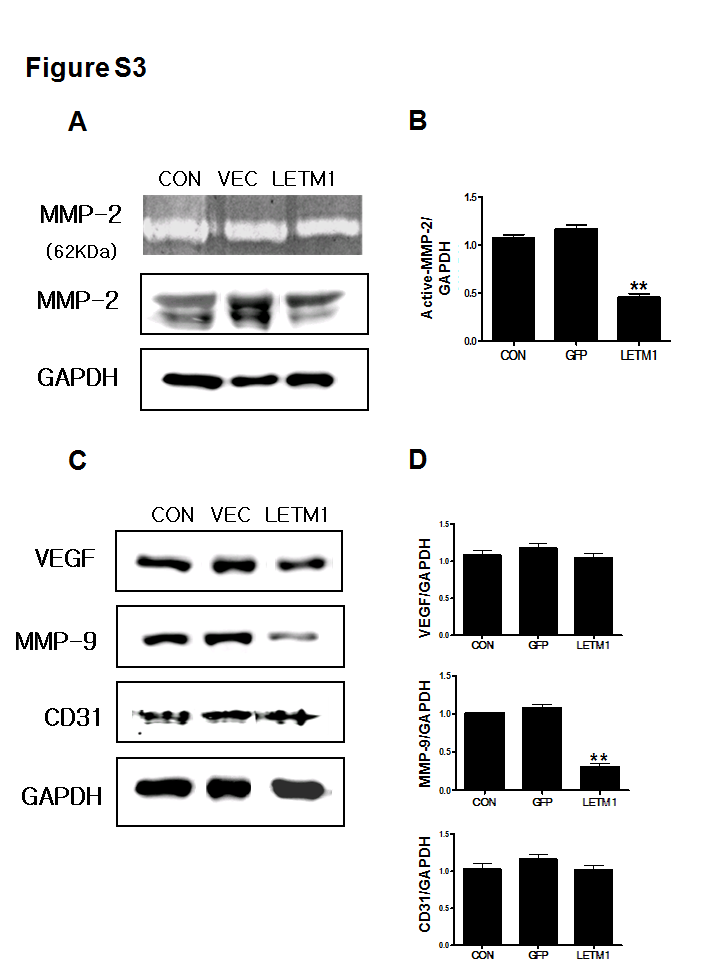

Supplement: Figure S3 — Immunohistochemistry of ki-67 and BCLxL in the lungs of K-ras LA1 mice. (A) Immunohistochemistry analysis of ki-67 in the lung. Dark brown color indicates the Ki-67 expression (magnification, X 100 and X400). (B) Comparison of ki-67 labeling index. ki-67 positive staining was determined by counting 10 randomly chosen fields per section, determining the percentage of DAB positive cell per 50 cells. (C) Immunohistochemistry analysis of BCLxL in the lung. Dark brown color indicates the BCLxL expression (magnification, X 100 and X400). (D) Comparison of BCLxL labeling index. BCLxL positive staining was determined by counting 10 randomly chosen fields per section, determining the percentage of DAB positive cell per 50 cells. Each bar represents mean±SE (n = 8), *P<0.05 was considered significant and **P<0.01 highly significant compared with corresponding control values. (0.42 MB TIF) [file pone.0012535.s003.tif]

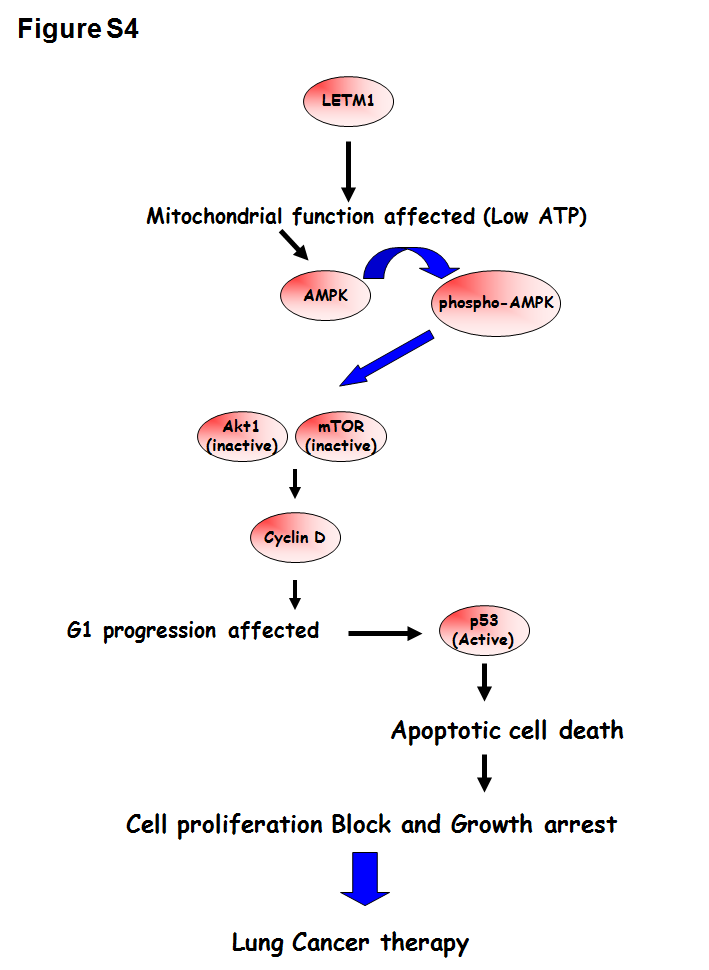

Supplement: Figure S4 — Schematic signal pathway by which LETM1 regulates lung cancer cell growth. LETM1 induced the activation of AMPK. Such activated AMPK inhibited Akt/mTOR signaling pathway and cyclinD1 expression level then decreased p53 and p21 expression level resulting in induction of apoptosis. (0.32 MB TIF) [file pone.0012535.s004.tif]
